# An investigation into the statistical precision attainable with a distribution-free method of constructing age-dependent reference centiles

**DOI:** 10.1371/journal.pone.0330330

**Published:** 2025-08-14

**Authors:** Stefan Wellek

**Affiliations:** 1 Department of Biostatistics, CIMH Mannheim, Mannheim Medical School of the University of Heidelberg, Mannheim, Germany; 2 Department of Medical Biostatistics, Epidemiology and Informatics, University Medical Center of the Johannes Gutenberg University Mainz, Mainz, Germany; Aichi Prefectural Mikawa Aoitori Medical and Rehabilitation Center for Developmental Disabilities, JAPAN

## Abstract

The distribution-free approach to the construction of age-dependent reference centiles which has been originally published by this author in 1995 and applied since then in a multitude of large-scale studies has never been investigated from a sample-size planning perspective. In the present paper, this gap is filled using the precision criterion introduced by Jennen-Steinmetz and Wellek (2005) for the estimation of reference centiles for quantitative diagnostic markers being independent of other variables, and extended by Jennen-Steinmetz (2014) to the study of age-dependent markers. In the age-dependent case, that criterion does not admit an exact representation as a function of the sample size, even when interest is in estimating a one-sided reference limit. Hence, all sample-size results presented here are based on Monte Carlo simulation. The computations cover a broad range of conditional distributions of the marker at given age including both symmetric and positively skewed distributions. For the relationship between the conditional standard deviation and age, a linear function of different slopes was assumed. Except for the most extreme settings investigated, the sample sizes shown in the tables summarizing our numerical results do not exceed the order of magnitude which has been available for a recent, potentially very influential reference-value study of basic parameters making-up the normal fetal growth profile. Furthermore, our results suggest that in terms of sample-size requirements, the distribution-free approach of Wellek & Merz (1995) to the construction of age-dependent reference ranges is typically a good bit more efficient than reference-range determination by means of quantile regression.

## 1 Introduction

By definition (see, e.g., [1–6]), reference limits for quantitative diagnostic markers are quantiles of the distribution followed by the respective random variable in the population of individuals free of the disease to be detected. Since as a whole, the latter is not accessible to observation, reference limits have in practice to be estimated from samples taken from that population. In the simplest case of a marker which does not depend on additional variables like age or body-weight, the sample consists of *n*  values being realizations of independent, identically distributed random variables $Y_{1},\ldots ,Y_{n}$ , say, with continuous cumulative distribution function (cdf) y↦F(y). The parametric estimates of the limits of a two-sided reference region with coverage (i.e., probability content) *q*  are given by

$$L_{n}=\bar{Y}-z_{(1+q)/2}S\;,\;U_{n}=\bar{Y}+z_{(1+q)/2}S,$$ (1)

where $\bar{Y}$ and S denotes, respectively, the mean and standard deviation of the *Y*_*i*_’s, and *z*_(1 + *q*)/2_ the upper 100(1+q)/2 –percentage point of the standard normal distribution. Nonparametrically, the estimated reference limits *L*_*n*_ and *U*_*n*_ are obtained through determining the order statistics of rank $r_{n}((1\;-\;q)/2)$ and $r_{n}((1\;+\;q)/2)$ defined as the smallest integer exceeding n(1−q)/2 and n(1+q)/2, respectively. Despite the simplicity of these estimators, a useful criterion of their statistical precision has been missing until the mid 2000s, where “useful” means in particular that it can be expressed in terms of a quantity which depends in a sufficiently regular way on the sample size *n*. The starting point of the approach developed by Jennen-Steinmetz & Wellek [7] for filling this gap is the fact that the crucial property of any estimator of a reference limit is the coverage it provides of the distribution of the diagnostic marker *Y*  in the underlying population of non-diseased individuals. None of the observed coverages *F*(*L*_*n*_) and *F*(*U*_*n*_) attained by estimating the individual limits of the reference interval should deviate from its target value (1−q)/2 and (1+q)/2, respectively, in either direction by more than some small tolerances $\delta_{1}$ and $\delta_{2}$. More precisely, the criterion requires that, when only one of both limits is thought to be of diagnostic relevance, the respective double inequality $(1-q)/2-\delta_{1}<F(L_{n})<(1-q)/2+\delta_{2}$ or $(1+q)/2-\delta_{1}<F(U_{n})<(1+q)/2+\delta_{2}$ holds with high probability *β* termed “confidence probability” in the sequel and to be computed under the assumption that the true distribution of *Y* is in fact *F*. In the two-sided case, i.e., when both “unusually” small and large values of *Y* are considered as possible disease indicators, *β* is defined to denote the probability of the joint event $\{(1-q)/2-\delta_{1}<F(L_{n})<(1-q)/2+\delta_{2}\}\cap \{(1+q)/2-\delta_{1}<F(U_{n})<(1+q)/2+\delta_{2}\}.$

Constructing reference centiles for an age-dependent quantitative diagnostic marker *Y* requires that the endpoints *L*_*n*_, *U*_*n*_ of a single interval be replaced with a couple *L*_*n*_(*t*), *U*_*n*_(*t*) of functions of time to be computed from a sample of pairs $\left(t_{1},Y_{1}),\ldots ,(t_{n},Y_{n}\right)$ with *t*_*i*_ denoting for each i=1,…,n, the age at which *Y*_*i*_ was measured. Roughly speaking, the distribution-free approach developed by Wellek & Merz [3] consists of determining *L*_*n*_(*t*), *U*_*n*_(*t*) in such a way that the band in the (*t*,*y*)-plane with the curves corresponding to these functions as boundaries, contains (almost) exactly a proportion of 100*q* percent of the data points $\left(t_{1},Y_{1}),\ldots ,(t_{n},Y_{n}\right)$ with equal proportions of 100((1−q)/2) percent falling above the upper and below the lower boundary curve, respectively. The approach proved in numerous applications a worthwhile alternative to the parametric methods proposed and refined by a number of authors (see [1,2,8–12]). Classing the procedure as distribution-free is justified by the fact that for each *t*, the conditional distribution function $F_{t}(\cdot )$ of *Y* is assumed to be of the form

$$F_{t}(y)=F((y-\mu (t))/\sigma (t)),$$ (2)

where μ(·) and σ(·) are continuous functions of time (age) and the baseline distribution-function F(·) is likewise assumed continuous but left fully unspecified otherwise. In the version being the object of the numerical investigations presented in this paper, the distribution-free construction of reference bands involves the following major steps.

STEP 1: Fitting by ordinary least-squares estimation a regression-function $t\mapsto \hat{\mu }(t)$  to the data  $\left(t_{1},Y_{1}),\ldots ,(t_{n},Y_{n}\right)$ which describes the relationship in the mean between *Y* and *t*  and yields a curve to be used as a pivotal line for the reference band.
STEP 2: Replacing the theoretical scale-factor function σ(·) by an estimate $\hat{\sigma }(\cdot )$ obtained through fitting regression line to the absolute residuals $\left|Y_{1}-\hat{\mu }(t_{1})|,\ldots ,|Y_{n}-\hat{\mu }(t_{n})\right|$.
STEP 3: Computing for the sample $\left((Y_{1}-\hat{\mu }(t_{1}))/\hat{\sigma }(t_{1}),\ldots ,(Y_{n}-\hat{\mu }(t_{n}))/\hat{\sigma }(t_{n})\right)$ of signed scaled residuals the order statistics of the same ranks *r*_*n*_((1−*q*)/2) and *r*_*n*_((1 + *q*)/2) as used for nonparametric estimation of the reference limits for an age-independent diagnostic marker.
STEP 4: Computing the boundaries of the reference band as
  $$L_{n}(t)=\hat{\mu }(t)+k_{l}(n,q)\hat{\sigma }(t),\;\;U_{n}(t)=\hat{\mu }(t)+k_{u}(n,q)\hat{\sigma }(t),$$ (3)
  where *k*_*l*_(*n*,*q*) and *k*_*u*_(*n*,*q*) are the order statistics obtained in STEP 3. (For a proper understanding of these equations, it is important to note that the coefficients *k*_*l*_(*n*,*q*) and *k*_*u*_(*n*,*q*) have opposite signs.)

The availability of a theoretically well-grounded method of sample-size planning is a basic desideratum also for studies aiming to establish reference values for diagnostic markers depending on patient’s age. Actually, dependence of a diagnostic marker on age (or some other quantitative covariate of the continuous type) does not alter the fact that reference limits are unsuitable for use in medical practice unless they are based on a study having included a sufficiently large number of individuals being free of the disease to be diagnosed. Relying on a sample of smaller size than necessary increases the risk of giving rise to a diagnostic test of a specificity which deviates unacceptably far from its target value (typically set at 95%). Including more individuals than necessary has likewise to be avoided, due both to ethical reasons and costs. The criterion proposed here for sample-size planning is conceptually analogous to the power in the context of a clinical trial to be analyzed by means of a test of significance for the hypothesis of primary interest.

**Fig 1:**
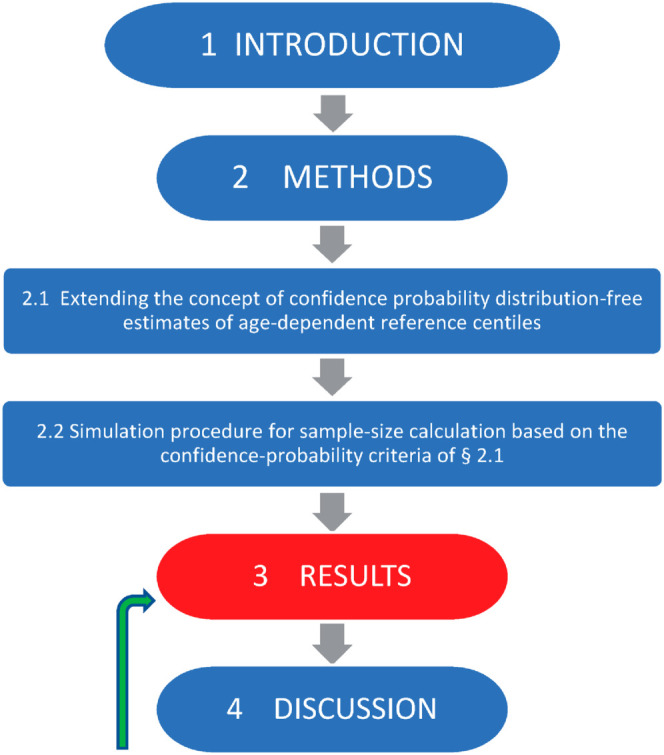
Flow-chart visualizing the organization of the body of the paper.

A flow-chart allowing for a quick glance over the organization of the body of the paper is shown in Fig 1.

## 2 Methods

### 2.1 Extending the concept of confidence probability to distribution-free estimates of age-dependent reference centiles

Conceptually, extending the definition of confidence probability made explicit in the Introduction for the age-independent case, to a precision criterion for reference bands constructed by means of the proposed distribution-free method is largely straightforward. One starts with evaluating at fixed time *t* the local precision of the interval $(L_{n}(t),\infty ),$ $\left(-\infty ,U_{n}(t)\right)$ and $(L_{n}(t),U_{n}(t)),$ respectively, taken as a reference range for the conditional distribution of *Y* given *T* = *t*. Subsequently, the least favorable of these local precision values is determined and used as a global precision criterion for the estimated limit(s) of the reference region as a whole. Considering once more the one-sided case first, the global confidence probabilities $\beta_{L}(n)$ and $\beta_{U}(n)$, say, are given by


$$\beta_{L}(n)=\min_{t\in 𝒯}{\mathbb{P}}_{Y|𝒯}^{\left(n\right)}[\{((1-q)/2-\delta_{1})<F((L_{n}(t)-\mu (t))/\sigma (t))<((1-q)/2+\delta_{2})\}]$$ (4.a)

and


$$\beta_{U}(n)=\min_{t\in 𝒯}{\mathbb{P}}_{Y|𝒯}^{\left(n\right)}[\{((1+q)/2-\delta_{1})<F((U_{n}(t)-\mu (t))/\sigma (t))<((1+q)/2+\delta_{2})\}].$$ (4.b)

In the above equations, ${\mathbb{P}}_{Y|𝒯}^{\left(n\right)}(\;\cdot \;)$ denotes the joint distribution (conditional on $\left(t_{1},\ldots ,t_{n})\in 𝒯\right)$ of the sample $\left(Y_{1},\ldots ,Y_{n}\right)$ from which *L*_*n*_ and *U*_*n*_ are to be calculated, and 𝒯 the (finite) set of points in time at which measurements of *Y* will be taken. In other words, the global confidence probability is defined by (4.a) and (4.b) as the smallest local confidence probability attained by $\left(L_{n}(t),\infty \right)$ and $\left(-\infty ,U_{n}(t)\right)$, respectively, in the conditional distribution of *Y* at any age *t* envisaged according to the time schedule of the study for taking measurements of *Y*. If interest is in assessing simultaneously the global precision to be attributed to both the lower and the upper boundary of the reference band, the sample size has to be chosen large enough to yield a sufficiently large value of the confidence probability $\beta_{\left(L,U\right)}(n)$ as defined by

$$\beta_{\left(L,U\right)}(n)=\min_{t\in 𝒯}{\mathbb{P}}_{Y|𝒯}^{\left(n\right)}[\{((1-q)/2-\delta_{1})<F((L_{n}(t)-\mu (t))/\sigma (t))<((1-q)/2+\delta_{2})\}\;\;\cap \{((1+q)/2-\delta_{1})<F((U_{n}(t)-\mu (t))/\sigma (t))<((1+q)/2+\delta_{2})\}].$$ (4)

### 2.2 Simulation procedure for sample-size calculation based on the confidence-probability criteria of Sect 2.1

Even for fixed *t*, the distribution of the statistics $F((L_{n}(t)\;-\;\mu (t))/\sigma (t))$, $F((U_{n}(t)\;-\;\mu (t))/\sigma (t))$ admits explicit representations only when 𝒯 consists just of a single point, i.e., in the age-independent case. In view of this, for computing the confidence probabilities of (4.a), (4.b) and (5) Monte Carlo simulation instead of exact integration with respect to ${\mathbb{P}}_{Y|𝒯}^{\left(n\right)}$ will be used throughout. The simulation scenarios to which the results reported in Sect 4 relate were designed to reflect basic features of two typical studies of reference centiles for quantities monitored in prenatal diagnostics.

**Fig 2:**
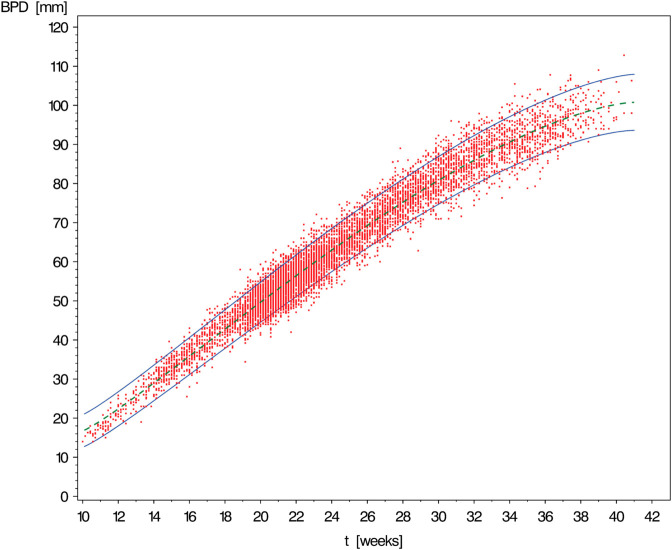
Biparietal diameter of n = 10225 fetuses by age of gestation [weeks] with 90% reference band constructed by the method under investigation in this paper (Data from Merz *et al*. [13]).

**Fig 3:**
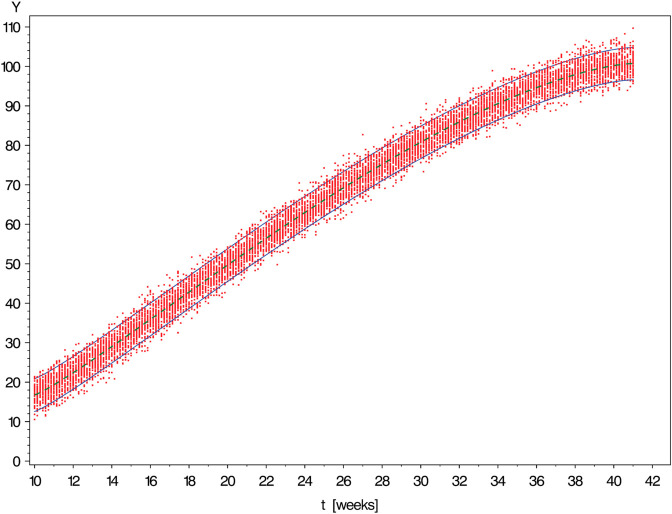
Simulated data set consisting of n = 10,000 observations generated from Scenario I with Gaussian conditional distributions of time-independent variance [↔ $\gamma_{1}=0$] and uniform distribution of measurement time (i.e., $a^{*}=b^{*}=1$). The solid curves are the limits of the estimated reference band of 2-sided target coverage *q* = 0.90.

**Fig 4:**
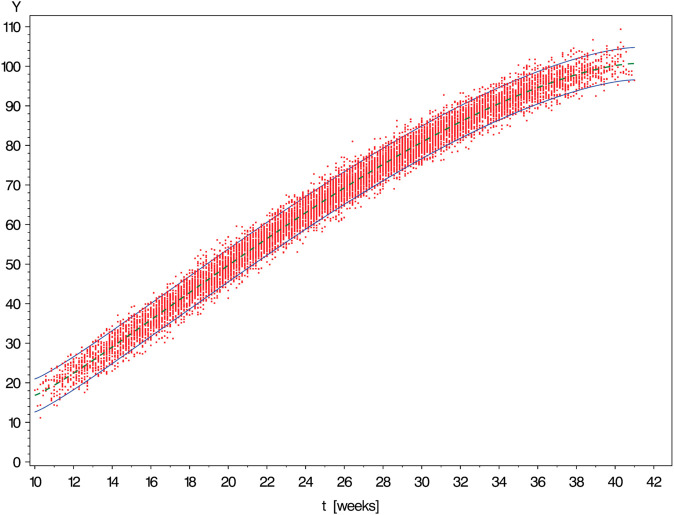
Simulated data set consisting of n = 10,000 observations generated from Scenario I with Gaussian conditional distributions of time-independent variance [↔ $\gamma_{1}=0$] and umbrella-shaped distribution of measurement time ($a^{*}=b^{*}=2$). The solid curves are the limits of the estimated reference band of 2-sided target coverage *q* = 0.90.

**Fig 5:**
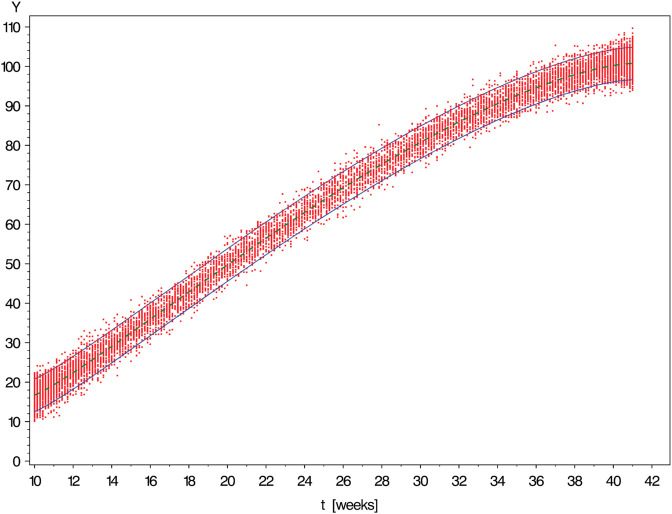
Simulated data set consisting of n = 10,000 observations generated from Scenario I with Gaussian conditional distributions of time-independent variance [↔ $\gamma_{1}=0$] and U-shaped distribution of measurement time ($a^{*}=b^{*}=0.5$). The solid curves are the limits of the estimated reference band of 2-sided target coverage *q* = 0.90.

**Fig 6:**
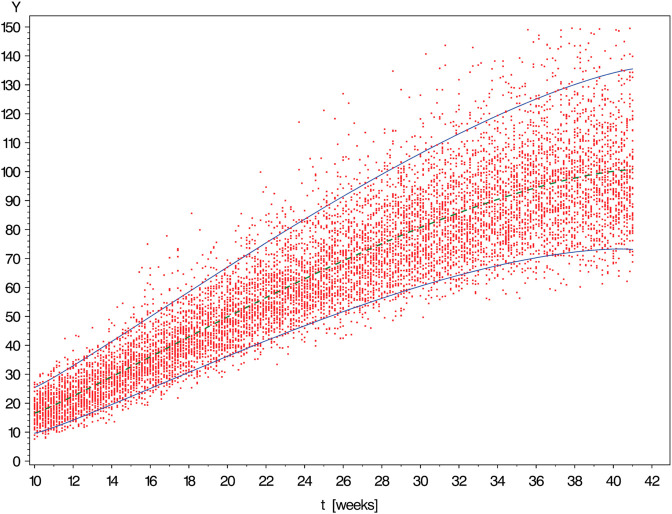
Simulated data set consisting of n = 10,000 observations generated from Scenario I with with log-normal conditional distributions of variance increasing in t [↔ $\gamma_{1}=3$] led uniform distribution of measurement time ($a^{*}=b^{*}=1$). The solid curves are the limits of the estimated reference band of 2-sided target coverage *q* = 0.90.

**Fig 7:**
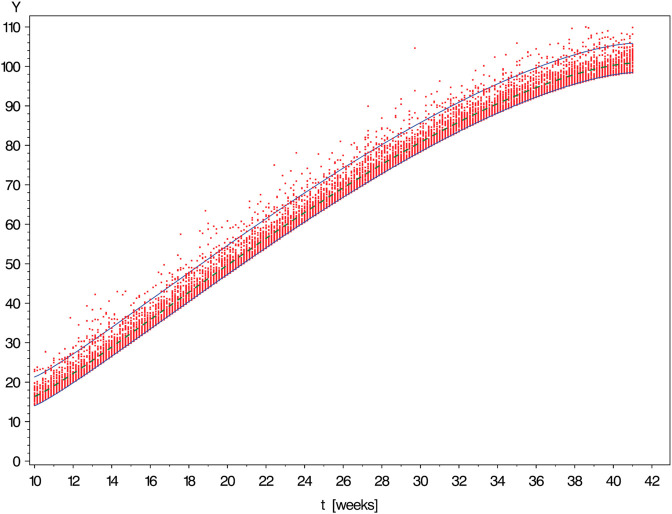
Simulated data set consisting of n = 10,000 observations generated from Scenario I with with conditional gamma distributions of time-independent variance [↔ $\gamma_{1}=1$] and uniform distribution of measurement time ($a^{*}=b^{*}=1$). The solid curves are the limits of the estimated reference band of 2-sided target coverage *q* = 0.90.

I The first scenario mimics some part of the study of Merz *et al*. [13] devoted to establishing age-dependent reference limits for quantities making up the fetal growth profile. The selected diagnostic marker is the biparietal diameter of the fetus for which the data and reference limits shown in Fig 2 were found in that study.
  For a proper understanding of this and all subsequent graphs, it is important to realize that due to limitations in measurement accuracy, the points of the cloud representing the raw data do not correspond throughout to individuals but small groups of varying size. Therefore, they do not allow for precise verification of the coverage proportion attained by the reference band. Nevertheless, the approximate symmetry of the conditional distributions obtained at different (gestational) ages becomes fairly conspicuous.
  The simulation scenario corresponding to the dataset shown in Fig 2 has been designed to meet the following specifications.
  i) *Timing of measurements:* 𝒯={10+j/7|0≤j≤217} [↔ set of gestational ages [weeks] between 10 and 41 inclusive]; ${𝕋}_{n}=(t_{1},\ldots ,t_{n})=$ vector of measurement times, fixed for all runs of a given Monte Carlo experiment and generated by drawing *n* values from a discretized beta distribution with parameters (${\text{a}}^{*},{\text{b}}^{*}$) on the set 𝒯. (In the special case $a^{*}=b^{*}=1$, ${𝕋}_{n}$ corresponds to a fixed uniform design.)
  ii) *Modelling the regression function μ(·):* The growth model proposed and extensively applied to data from prenatal ultrasound by Wellek & Merz [14] was used. This model assumes that their holds $\mu (t)=c\;(d\;+\;I_{\left(t-t^{\prime })/(t^{\prime \prime }-t^{\prime }\right)}(a,b))$ where a,…,d are unknown parameters to be estimated using the data, and $\left[t^{\prime },t^{\prime \prime }\right]$ and $I_{\;\cdot \;}(a,b)$, respectively, denotes the range of *t* (set equal to [10,41] under the specifications made in (i)) and the cdf of a beta distribution with parameters *a*  and *b*. In generating the data, it was assumed that the estimates *a* = 1.1494, *b* = 1.5265, *c* = 84.0244, *d* = 0.1992 obtained in the study from which Fig 2 is taken, are the true values of the parameters of the growth model.
  iii) *Modelling the dispersion function σ(·):* Throughout, σ(t) was chosen to be a linear function of t with parameters $\gamma_{0}$, $\gamma_{1}$ to be determined by way of regressing on *t* the absolute residuals obtained through fitting μ(·) to the respective simulated sample $\left(t_{1},Y_{1}),\ldots ,(t_{n},Y_{n}\right)$.
  iv) *Specifying the baseline cdf F(·) of the conditional distribution of (Y−μ(t))/σ(t) at age t:* The following basic forms of the age-specific distributions were investigated:
    a) Gaussian [↔F(t)=Φ(t)]
    b) Laplace [↔F(t)=(1/2)(1+sign(t)(1−exp(−|t|)))]
    c) Log-normal $\left[↔F(t)=\Phi ((\log(t+\exp(\mu_{0}+\sigma_{0}^{2}/2)))/\sigma_{0})\right]$
    d) Gamma $\left[↔F(t)=\Gamma_{\alpha }(t/\lambda_{0}+\sigma_{0})\right]$.
    Note. All these distributions have mean zero.
  Figs 3, 4, 5, 6, and 7 show simulated data sets consisting of n=10,000 observations generated from different versions of Scenario I together with the estimated reference band of 2-sided target coverage *q* = 0.90 each.
The population from which the simulated dataset shown in Fig 3 has been taken, is a particularly regular case within the scope of Scenario : All conditional distributions satisfy a model which differs from a classical linear model only by the nonlinear form of the regression function. Furthermore, the distribution of age is uniform over the range [10,41] (weeks).
  The only although crucial difference between the models underlying the above figure and Fig 3 is that this time the distribution of gestational age is umbrella-shaped instead of uniform.
  In the example of Fig 5, the conditional distributions are likewise the same as in Fig 2. However, sampling was done in a way ensuring that both extreme tails of the distribution of age are distinctly heavier than a central interval of the same length.
  Under the log-normal model, the variance is proportional to the mean implying that the reference band shown in Fig 6 increases in vertical width from left to right.
  Comparing Fig 7 with the previously shown diagram one finds it corroborated that both the lognormal and the gamma distributions are skewed to the right. With the parameters set to the values chosen in both examples, this skewness is distinctly more marked for the gamma as compared to the lognormal model.
II The other scenario was constructed referring to the reference band presented by Merz & Pashaj [15] for the frontal fetal facial angle (FFFA) as measured by 3D ultrasound. The crucial difference as compared with Scenario I concerns the form of the regression function μ(·) which is now assumed to be a polynomial of 4th degree. For its value at age *t* we write $\mu (t)=a_{0}\;+\;a_{1}t\;+\;a_{2}t^{2}\;+\;a_{3}t^{3}\;+\;a_{4}t^{4}$. In the simulations using Scenario II, the data were generated setting $a_{0}=275.548,a_{1}=-19.4763,a_{2}=1.00317,a_{3}=-0.021839,a_{4}=\mathrm{.000171349}$. (These are the estimates obtained by Merz & Pashaj). The range 𝒯 of ages *t* of gestation [days] at which measurements of FFFA are taken in that study is assumed to be {t∈N|77≤t≤280}

As software, SAS/IML was used throughout, generating separate single data streams for all repetitions of the same Monte Carlo experiment. The target of all simulation studies performed was the sample size *n*  required for ensuring that the confidence probability in one of the definitions given in (4.a), (4.b) and (5) does not fall short of $\beta_{0}=90\%$. Throughout,the tolerances $\delta_{1}$ and $\delta_{2}$ upon which the confidence probabilities depend where chosen to be 0.02. With a view to the inherent limitations regarding the exactness of simulated values of the probabilities used in the present context as criteria of statistical precision, all sample sizes have been determined as multiples of 10. For scenarios with a polynomial regression of *Y* on *t*, the results were compared to those obtained by Jennen-Steinmetz [16] for reference regions constructed by means of quantile regression in the sense of Koenker [17].

## 3 Results

A complete account of the sample sizes calculated for Scenario I with a variety of specifications of the parameters involved is given in Table 1.

**Table 1:** Simulated sample sizes required for attaining confidence probability β=90% in Scenario I. [Target coverage q=0.90 ; tolerances $\delta_{1}=\delta_{2}=0.02$.]

Parameters of Age Distrib.		Form of the Conditional Distributions of *Y*	Slope of σ(t)	Sample Sizes		
*a* ^*^	*b* ^*^			$n_{L}$	$n_{U}$	$n_{\left(L,U\right)}$
1.00	1.00	**Gaussian**	0.0000	2110	2110	3180
"	"	"	0.0817	2950	2950	4310
"	"	"	0.2450	5490	5490	7790
1.50	1.50	"	0.0000	4310	4310	5960
2.00	2.00	"	"	6910	6910	10210
"	"	"	0.0817	10930	10930	14480
"	"	"	0.2450	23310	23310	32010
0.75	0.75	"	0.0000	1610	1610	2260
0.50	0.50	"	"	1160	1160	1620
"	"	"	0.0817	1380	1380	1920
"	"	"	0.2450	2040	2040	2890
1.00	1.00	**Laplace**	0.0000	1390	1390	1890
"	"	"	0.0817	1730	1730	2680
"	"	"	0.2450	2960	2960	4830
1.50	1.50	"	0.0000	2310	2310	3300
"	"	"	0.0817	3050	3050	4820
2.00	2.00	"	0.0000	3780	3780	5170
"	"	"	0.0817	5580	5580	8090
"	"	"	0.2450	12170	12170	17510
0.75	0.75	"	0.0000	1040	1040	1470
0.50	0.50	"	"	810	810	1110
"	"	"	0.0817	930	930	1350
"	"	"	0.2450	1370	1370	2020
1.00	1.00	**Log-Normal**	0.0000	4440	1480	4480
"	"	"	0.0817	6210	1960	6240
"	"	"	0.2450	11820	3570	11930
1.50	1.50	"	0.0000	8990	2490	8990
2.00	2.00	"	"	15460	4020	15460
"	"	"	0.0817	24730	6490	24730
"	"	"	0.2450	53790	12890	53790
0.75	0.75	"	0.0000	2880	1120	2970
0.50	0.50	"	"	1820	880	1960
"	"	"	0.0817	2380	1010	2540
"	"	"	0.2450	3910	1510	4220
1.00	1.00	**Gamma**	0.0000	4090	1680	4170
"	"	"	0.0817	5770	2120	5840
"	"	"	0.2450	10970	3870	11380
1.50	1.50	"	0.0000	8470	2820	8470
"	"	"	0.0817	12130	3920	12420
2.00	2.00	"	0.0000	14730	4580	14730
"	"	"	0.0817	22900	6920	22900
"	"	"	0.2450	49300	14380	49300

The results show that the sample-size requirements for a reference-value study to be performed in this scenario heavily depends both on the form of the conditional distributions, the dispersion function and the pattern 𝒯 of measurement times. The most obvious and consistent relationship between one of these properties of the study design and the required sample size *n*  has to be attributed to the slope of the dispersion function chosen to be given by the parameter $\gamma_{1}$. Given anything else, *n* is a monotonically increasing function of $\gamma_{1}$. The steepness of this increase is considerable indeed. The factor by which the sample size has to be multiplied when a constant dispersion function is replaced with a linear function of slope 0.2450 depends on all other parameters varied across the simulation experiments and may be as large as about 3.5. Effects which are fully uniform in all other parameters result from varying the pattern of measurement times: Choosing the common value of the parameters (${\text{a}}^{*},{\text{b}}^{*}$) as a number >1.0, the values of *T* are taken from a discretized symmetric unimodal density, implying that the proportion of the observations made available for ages in the tails of the distribution of *T* are scarce which leads to increased values of *n*. Using designs with *U*-shaped distribution of *T* letting both components of the parameter (${\text{a}}^{*},{\text{b}}^{*}$) fall below unity so that the distribution of *T* exhibits heavy tails, reduces the sample sizes below those required for a uniform design.

For right-skewed conditional distributions, the sample size required for estimating at a given level of precision the limits of a one-sided reference region are considerably larger when the estimand is a lower bound as compared with the corresponding right-hand reference limit. The amount of skewness has a marked increasing effect on the sample sizes required for estimating lower reference limits, then. In contrast, the sample sizes needed for estimating upper reference limits tend to be smaller for distributions with positive skewness as compared with symmetric distributions. In symmetric cases, *n*_*L*_ and *n*_*U*_ coincide except for simulation errors which is in accordance with intuition. Furthermore, the sample sizes *n*_(*L*,*U*)_ required for studies aiming to estimate a two-sided reference region are a good bit larger than the sample sizes needed for estimating one-sided reference limits, exceeding the latter by up to 63% [→ Laplacian distribution for a uniform design with slope 0.2450 of the dispersion function]. In contrast, when the distributional shape exhibits skewness on the right, *n*_(*L*,*U*)_ is only slightly larger than the sample size *n*_*L*_ for the corresponding left-hand estimation problem.

Even between distributions of the symmetric type, there are marked differences: With Gaussian conditional distributions, the sample-size requirements are uniformly considerably larger as compared with their Laplacian counterparts. Some of the differences exceed the 100% -bound [→ umbrella-shaped design with $\left(a^{*},b^{*})=(2.0,2.0\right)$ and slope 0.2450 of σ(t)]. The comparisons between the lognormal and the gamma distribution do not reveal an uniform pattern: There are both constellations for which a study of an age-dependent lognormally distributed diagnostic marker requires larger sample sizes than a gamma-distributed marker as well as instances where this difference has the opposite sign.

The differences between homologous entries in Tables 1 and 2 are moderate. This is not surprising since the regression models according to which data generation is carried out in Setting I and II are of comparable complexity. In terms of the dimension of the unknown parameters to be estimated from the data, both cases differ just by unity. Computationally, the second scenario is a good bit easier to handle than Scenario I since in the latter the estimates of the parameters of the regression function must be determined by means of an iteration algorithm instead of explicit formulae.

**Table 2:** Simulated sample sizes required for attaining confidence probability β=90% in Scenario II. [Target coverage q=0.90 ; tolerances $\delta_{1}=\delta_{2}=0.02$.]

Parameters of Age Distrib.		Form of the Conditional Distributions of *Y*	Slope of σ(t)	Sample Sizes		
*a* ^*^	*b* ^*^			$n_{L}$	$n_{U}$	$n_{\left(L,U\right)}$
1.00	1.00	**Gaussian**	0.0000	2350	2350	3330
"	"	"	0.2620	2960	2960	4220
"	"	"	0.7860	5060	5060	7400
1.50	1.50	"	0.0000	4490	4490	6170
2.00	2.00	"	"	7990	7990	10990
"	"	"	0.2620	11420	11420	15170
"	"	"	0.7860	22140	22140	30460
0.75	0.75	"	0.0000	1610	1610	2310
0.50	0.50	"	"	1140	1140	1590
"	"	"	0.2620	1410	1410	1850
"	"	"	0.7860	1890	1890	2760
1.00	1.00	**Laplace**	0.0000	1330	1330	1950
"	"	"	0.2620	1770	1770	2620
"	"	"	0.7860	3010	3010	4410
1.50	1.50	"	0.0000	2450	2450	3500
"	"	"	0.2620	3340	3340	4830
"	"	"	0.2450	6190	6190	9070
2.00	2.00	"	0.0000	4220	4220	5950
"	"	"	0.2620	5960	5960	8570
"	"	"	0.7860	11730	11730	16790
0.75	0.75	"	0.0000	1030	1030	1510
0.50	0.50	"	"	790	790	1130
"	"	"	0.2620	950	950	1330
"	"	"	0.7860	1340	1340	1930
1.00	1.00	**Log-Normal**	0.0000	4590	1540	4640
* "	"	"	0.2620	6110	1940	6260
* "	"	"	0.7860	11070	3170	11320
* 1.50	1.50	"	0.0000	9790	2610	9790
2.00	2.00	"	"	18270	4510	18270
"	"	"	0.2620	27080	6250	27080
"	"	"	0.7860	53790	12110	53790
0.75	0.75	"	0.0000	2940	1210	3120
0.50	0.50	"	"	1810	890	2000
"	"	"	0.2620	2300	1010	2510
"	"	"	0.7860	3730	1410	4010
1.00	1.00	**Gamma**	0.0000	4230	1710	4400
"	"	"	0.2620	5620	2130	5970
"	"	"	0.7860	10140	3370	10140
1.50	1.50	"	0.0000	9180	3010	9180
"	"	"	0.2620	12640	3880	12420
2.00	2.00	"	0.0000	17240	4980	17240
"	"	"	0.2620	24370	6990	24370
"	"	"	0.7860	49530	13330	49530
0.75	0.75	"	0.0000	3010	1210	3010
0.50	0.50	"	"	1810	1010	1930
"	"	"	0.2620	2210	1060	2350
"	"	"	0.7860	3340	1510	3660

## 4 Discussion

A natural competitor to the distribution-free method of constructing age-dependent reference centiles investigated in this paper is quantile regression (QR) as introduced by Koenker [17] .

The paper by Jennen-Steinmetz [16] on sample-size determination for studies aiming to establish reference centiles for age-dependent quantitative diagnostic markers covers also the QR-based approach, restricting the model to be used for describing the relationship of the marker on time to polynomials of second degree at most. Clearly, in any given setting and based on the same criterion of precision, estimating a reference centile curve when the true underlying regression on age is a quadratic polynomial, requires considerable lower sample sizes as compared with a marker depending on time according to polynomial regression of degree 4.

Furthermore, in Scenario II, for a uniform design, i.e., for $\left(a^{*},b^{*})=(1.00,1.00\right)$, choosing the slope of the dispersion function σ(t) to be equal to 0.0000, 0.2620 and 0.7860 [cf. Column 4 of Table 2] is equivalent to setting in the tables of Jennen-Steinmetz [16] $\gamma_{1}$ equal to 0, 1 and 3, respectively. Thus, the entries in Columns 10 and 12 of Line 12 of Jennen-Steinmetz’ Table 2 can be directly compared to the values of *n*_*L*_ and *n*_*U*_ shown in the upper three lines of our Table 2. Although they hold for a setting with quadratic rather than polynomial regression of degree 4, the former are substantially larger than the latter (≈2900 vs <2200 and ≈3400 vs <3000 for a σ(t) with slope 0.0000 and 0.2620, respectively) as long as the conditional distributions are symmetric. Even when F(·) is markedly right-skewed, the sample sizes required for our approach exceed those for the QR-approach only when interest is in estimating a lower reference limit whereas for estimation of an upper limit the sign of the difference between the respective sample sizes is reverse. Overall, these comparisons admit the conclusion that the estimation procedure studied here is in many cases considerably more efficient than quantile regression. In addition, the QR approach suffers from the serious disadvantage that it produces reference bands whose upper boundary may differ in shape from its lower counterpart.

Finally, it is important to note that the majority of entries in Table 1 do not exceed 10,000. This is the order of magnitude of the sample size which has been available for a recent, potentially highly influential reference-value study of basic parameters making-up the normal fetal growth profile (Merz *et al*. [13]). (Some parts of the results of a previous version were included in the Mother’s Passport delivered by the GBA [Joint Federal Committee of Germany] to every pregnant woman as a tool for monitoring the course of pregnancy.) This suggests that even under marked asymmetry and/or heteroscedasticity of the underlying conditional distributions, the age-dependent reference centiles published in that source satisfy fairly rigorous criteria of estimation precision.

All numerical results obtained in the simulation study hold under the restriction that the dispersion function is linear in age *t*. Admitting more complicated models for σ(t) is a conceptually straightforward generalization but has to be expected to lead to sample sizes which are still larger than those obtained under the assumption of linearity. Fortunately, extensive practical experience has shown that in real studies of age-dependent diagnostic markers, marked deviations from dispersion linearity rarely occur. The adequacy of the assumption can readily checked through analyzing the absolute residuals calculated for the nonlinear regression model for the means. The regression of these residuals on age should be approximately linear.

As holds true for any numerical investigation using Monte Carlo methods, the results obtained in this paper are not fully exact but overlaid by (small) simulation errors. Evidently, in the present context the relevant target of an analysis of these errors is the standard error of the simulated confidence probability (defined as in Equations (4.a, b) and (5) ) attainable with one of the sample sizes *n* to be read from Table 1 or Table 2. Since the aim was to determine *n* as large as necessary for ensuring that under the respective specifications the confidence probability reaches the value 90% at least, and the number of replications was set at *N*_*rep*_ = 10,000 for the majority of the Monte Carlo experiments behind Tables 1 and 2, an upper bound to the estimated Monte Carlo standard error of the simulated value of $\beta_{L}(n)$, $\beta_{U}(n)$, and $\beta_{L,U}(n)$, respectively, is given by ${\text{stderr}}^{*}=\sqrt{\mathrm{.90}\cdot (1-\mathrm{.90})/10^{4}}$ = 0.003. This yields approximate 95-confidence intervals for the true values of $\beta_{L}(n)$, $\beta_{U}(n)$ and $\beta_{L,U}(n)$ associated with the tabulated sample sizes *n* whose length is less than 0.012 guaranteeing high precision of those pivotal estimates. In some cases, the entries in Tables 1 and 2 could be even based on 40,000 repetitions per Monte Carlo experiment which reduces ${\text{stderr}}^{*}$ to 0.0015 and the upper bound to the length of an approximate 95-confidence interval for $\beta_{L}(n)$, $\beta_{U}(n)$, and $\beta_{L,U}(n)$ to 0.006.

A particularly promising way of exploiting the results obtained in this paper for improving diagnostic decision making in various medical disciplines would be to screen the literature on reference-limit studies analyzed by means of the distribution-free method of constructing age-dependent centiles investigated here for compatibility with the sample-size criteria to be read from the entries in the tables shown above. Wherever it turns out that the sample sizes which were actually made available fell substantially short of the values required according to that criteria, a recommendable step would consist of planning a replication study set to recruit a reference sample of an appropriately increased size.
